# Ustekinumab merits further exploration in Takayasu arteritis despite a failed randomized controlled trial

**DOI:** 10.1093/rap/rkaf038

**Published:** 2025-03-25

**Authors:** Durga Prasanna Misra

**Affiliations:** Department of Clinical Immunology and Rheumatology, Sanjay Gandhi Postgraduate Institute of Medical Sciences (SGPGIMS), Lucknow, India

Takayasu arteritis (TAK) is a rare large vessel vasculitis associated with considerable morbidity and mortality [1, 2]. Despite the immune-mediated arterial wall damage that underlies the pathology of TAK, high-quality evidence for its treatment with immunosuppressive agents is lacking [1, 3]. A randomized controlled trial (RCT) evaluating abatacept in patients with TAK showed similar relapse-free survival compared with placebo. Another RCT assessed the efficacy of tocilizumab versus placebo to prevent relapses in patients with TAK. The trial failed to meet its primary end-point of the superiority of tocilizumab using an intention-to-treat analysis. However, a secondary per-protocol analysis demonstrated the benefit of tocilizumab over placebo [1]. An RCT of ustekinumab in patients with relapsing TAK was recently published in the journal, which failed to demonstrate a benefit with ustekinumab over placebo [4]. This editorial discusses this RCT in the context of the pathogenesis of TAK.

The role of T lymphocytes in driving the pathogenesis of arterial wall inflammation and damage in patients with TAK is well recognized. T helper (Th) 1 and Th17 lymphocytes drive granulomatous arterial wall inflammation, whereas Th17 lymphocytes contribute to arterial fibrosis and damage in patients with TAK [5, 6]. Th17 lymphocytes have been associated with disease activity in patients with TAK [5, 6]. The cytokine IL-12 drives the polarization of naïve T-helper lymphocytes to Th1 lymphocytes, whereas IL-23 maintains the differentiated population of Th17 lymphocytes. The major sources of IL-12 and IL-23 are macrophages and dendritic cells. The common p40 subunit transcribed from the *IL12B* gene locus is shared by IL-12 (a heterodimer of p35 and p40, together also known as IL-12p70) and IL-23 (a heterodimer of p19 and p40) (Fig. 1). Genome-wide association studies have identified the association of single-nucleotide polymorphisms in the *IL12B* gene locus with susceptibility towards TAK [7]. Homozygosity or heterozygosity for the A allele in rs6871626, a single nucleotide polymorphism in the *IL12B* gene locus, has been associated with higher circulating Th1 lymphocytes and increased levels of IL-12p70 in patients with TAK. Cultured monocytes from these individuals also secreted higher levels of IL-12p40 and IL-12p70 [8]. Homozygosity or heterozygosity for the A allele in rs6871626 was also associated with higher damage scores using either the Vasculitis Damage Index or the Takayasu Arteritis Damage Score in patients with TAK even after adjusting for the duration of disease [9]. Therefore, there is a strong rationale for evaluating the targeting of IL-12p40 for treating TAK.

**Figure 1.**
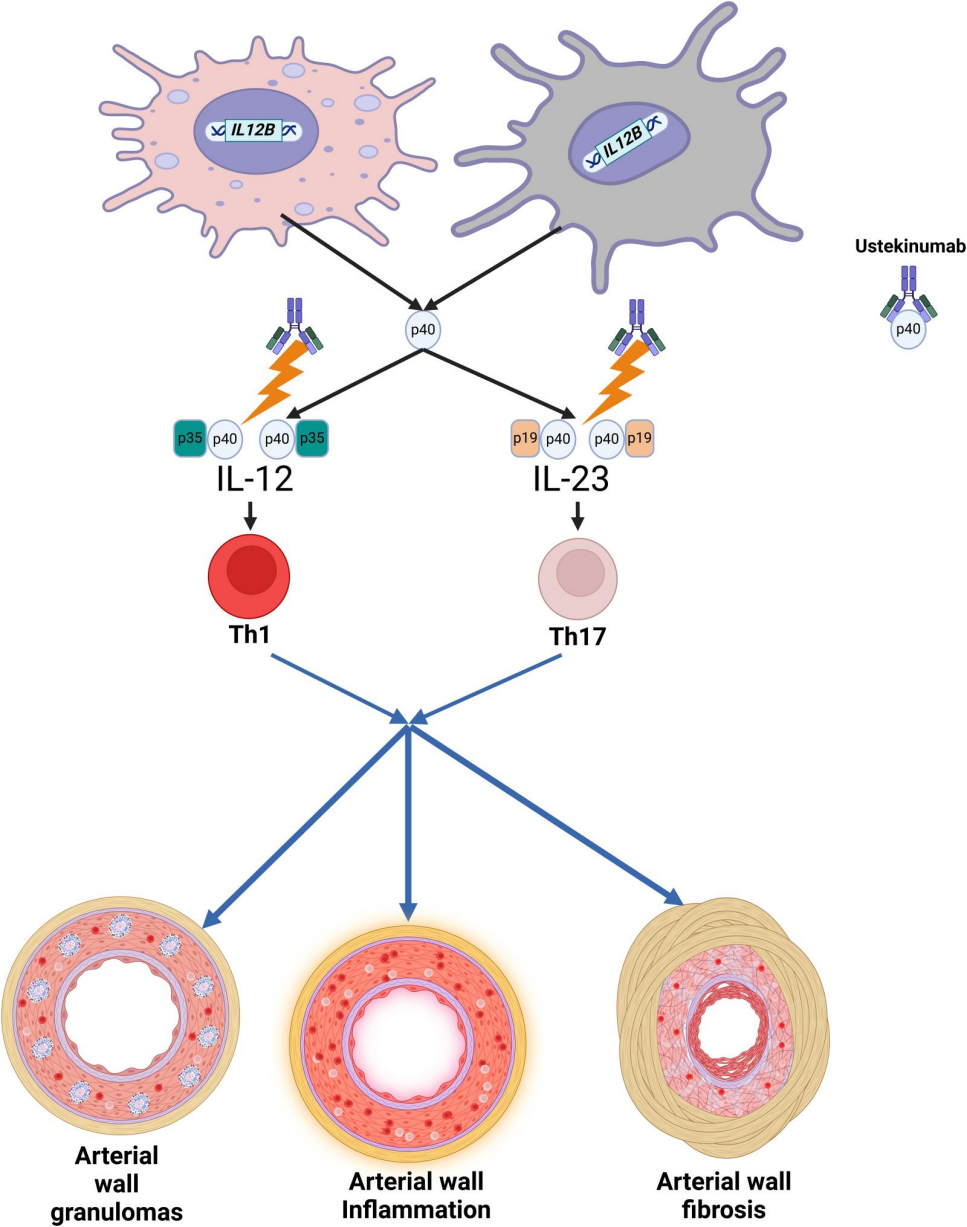
Rationale for the potential utility of ustekinumab in Takayasu arteritis. Created in https://BioRender.com/e12d914

Ustekinumab is a fully human IgG1 monoclonal antibody targeted to the common p40 subunit of IL-12 and IL-23, which has been successfully used in other rheumatic diseases as well as in ulcerative colitis (a disease that has been associated with TAK). The double-blind, placebo-controlled RCT of ustekinumab in patients with relapsing TAK, recently published in the journal, compared eight patients with TAK treated with ustekinumab with six treated with placebo [4]. No differences in the primary endpoint of relapse-free survival were observed between ustekinumab and placebo. However, the trial had to be terminated prematurely due to poor recruitment exacerbated by the timing of the initiation of the trial during the Coronavirus disease 19 (COVID-19) pandemic and did not meet its target of recruitment of 25 patients in each group. The trial used a customized definition of relapses based on the presence of any two from subjective or objective constitutional features of disease activity, elevation of acute-phase reactants, ischaemic symptoms or features of vascular inflammation [4]. Trials of patients with TAK have used various definitions of relapse. It might have been better to use a standard definition of relapse as proposed by the EULAR recommendations for the management of large vessel vasculitis. These define major relapses as the reappearance of active disease along with either ischaemic manifestations (such as stroke, ocular ischaemia, limb claudication) or angiographic evidence of progressive large vessel inflammation resulting in stenosis, dilatation or arterial dissection. Minor relapses are defined as the reappearance of active disease that does not meet the criteria for a major relapse. Of note, the elevation of acute phase reactants without supporting clinical features does not indicate active disease in patients with TAK [10]. The relapse-based endpoint used in the trial was problematic, as relapses are unpredictable. Therefore, such an endpoint might result in an underpowered trial. The trial included patients with TAK who had experienced a relapse in the past 12 weeks and thereafter achieved remission after treatment with glucocorticoids. The patient population was heterogeneous, with some already treated with biologic DMARDs [4]. Including a treatment-naïve population of patients with TAK (as opposed to relapsing patients) might have enhanced study recruitment. This trial also did not assess serial changes in angiography, which are recognized as important outcomes in patients with TAK. Given the association of the rs6871626 polymorphism in the *IL12B* gene locus with damage in patients with TAK, it might have been prudent to include clinical and angiographic measures of arterial wall damage as end-points in this trial. The lack of assessment of any patient-reported outcomes, which are increasingly recognized as important in TAK and other rheumatic diseases, was another limitation of this trial.

Given the limitations of the present RCT in design (particularly the narrow inclusion criteria) and the challenges in its execution from sub-optimal trial recruitment, further RCTs are warranted to evaluate the efficacy of ustekinumab in patients with TAK. Such trials should include a serial assessment of angiographic damage quantified using available scoring systems at least a year apart, as well as validated damage scores such as the Vasculitis Damage Index, as key endpoints [1].

## Data Availability

All the analyses performed for this article have been reported in the main text or in the supplementary files. Data pertaining to the article shall be shared on reasonable request to the corresponding author.
